# Management of acute Achilles tendon ruptures: a survey of Army orthopaedic surgeons

**DOI:** 10.1186/s12891-021-04121-y

**Published:** 2021-03-11

**Authors:** Nischal Nadig, Thomas Dowd, Jeannie Huh

**Affiliations:** 1 Department of Orthopaedic Surgery, Dwight D. Eisenhower Army Medical Center, 300 E Hospital Rd, Fort Gordon, GA 30905 USA; 2 Department of Orthopaedics & Rehab., San Antonio Military Medical Center, 3551 Roger Brooke Dr, San Antonio, TX 78219 USA; 3 Department of Orthopaedics & Rehab., Womack Army Medical Center, 2817 Reilly Rd, Fort Bragg, NC 28310 USA

**Keywords:** Achilles rupture, Acute rupture, United states army, Tendon rupture

## Abstract

**Background:**

Despite the literature on acute Achilles tendon ruptures, there remains a lack of consensus regarding the optimal treatment. The purpose of this survey study was to investigate treatment preferences among Army orthopaedic surgeons when presented with a standardized case of an acute Achilles rupture and determine if surgeon factors correlated with treatment preference.

**Methods:**

A hypothetical case of a 37-year-old male with history, physical exam, and imaging consistent with an Achilles rupture was sent to board-certified Army orthopaedic surgeons to determine their preferred management. Demographic data was collected to include: practice setting, years from residency graduation, and completion of fellowship. Correlations analyzed between demographics and treatment preferences.

**Results:**

Sixty-two surgeons responded. 62% of respondents selected surgical intervention. Of these, 59% chose a traditional open technique. 50% of respondents were general orthopaedic. There was a correlation between fellowship training and operative management (*P* = 0.042). Within the operative management group there was no statistical difference (*P* > 0.05) in need for further imaging, technique used, post-operative immobilization, length of immobilization, weight-bearing protocol, and time to release to running. The majority of non-operative responders would splint/cast in plantarflexion or CAM boot with heel lift for < 3 weeks (50%) and keep non-weight bearing for < 4 weeks (63%). Only 38% of respondents would use DVT chemoprophylaxis.

**Conclusion:**

When provided with a hypothetic case of an acute Achilles tendon rupture, queried Army orthopaedic surgeons would more often treat with a surgical procedure. This difference in treatment is secondary to training, fellowship or other. This propensity of surgical management, likely stems from the highly active population and the desire to return to duty.

**Supplementary Information:**

The online version contains supplementary material available at 10.1186/s12891-021-04121-y.

## Background

To date, there remains controversy on the optimum treatment for acute Achilles tendon ruptures. Operative treatment has been associated with decreased re-rupture rates, improved heel-rise strength, and earlier return to work as compared to non-operative treatment [1–4]. During a traditional open surgical approach, an incision made over the ruptured tendon and the tendon ends sutured together to allow for direct healing, however this places the patient at a potential for infection, blood loss, nerve damage and wound dehiscence. Over the years, techniques requiring smaller incisions and less invasive procedures have been devised to allow for a robust repair while also decreasing the risk of wound complications [5].

With the advent of the functional rehabilitation protocol, originally published by Willits et al [6], results similar to operative management have been demonstrated [6–11]. The accelerated rehabilitation protocol as detailed in Willits’ study [6] entails the patient being placed into a posterior slab splint in maximum plantarflexion for 2 weeks at time of injury. At the two-week mark, the patient is transitioned into a walking boot with a 2-cm heel lift and allowed to be weight-bearing as tolerated with crutches for assistance. One centimeter is removed every 2 weeks from the heel lift, until 8 weeks, at which point the patient begins to wean out of the boot. Non-operative management eliminates exposure to surgical complications such as infections (deep and superficial), blood loss, wound dehiscence, and risks associated with anesthesia [12, 13].

Few studies have examined this debate in the military population, which represents a young, athletic cohort nearly a decade younger than those of other studied groups [14, 15]. The purpose of this study was to survey the current management preferences of Army orthopaedic surgeons for acute Achilles tendon ruptures. Our hypothesis is that Army orthopaedic surgeons prefer to surgically manage acute Achilles ruptures.

## Methods

Eisenhower Army Medical Center Institutional Review Board determined “exempt” status for this study, as defined by the Federal Regulations for Protected Human Research Subjects, due to very minimal or no risk to patients. Respondents provided consent at time of responding to the survey. A current list of United States (US) Army orthopaedic surgeons was obtained from the orthopaedic consultant to the Surgeon General. The inclusion criteria for subjects to be surveyed included active duty Army orthopaedic surgeons who treated Achilles tendon ruptures in their clinical practice. Subjects were excluded if they were in a training program (residency or fellowship) or responded that do not treat Achilles tendon ruptures. The following surgeon characteristics were collected: years in practice, work setting, and whether or not they had fellowship training, including type of fellowship training. A total of 143 surgeons met inclusion criteria and 62/143 completed the survey, a response rate of 43%.

**Fig. 1:**
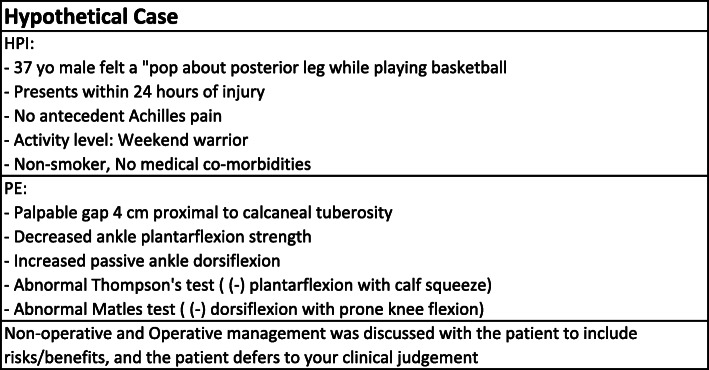
Hypothetical Case

**Fig. 2:**
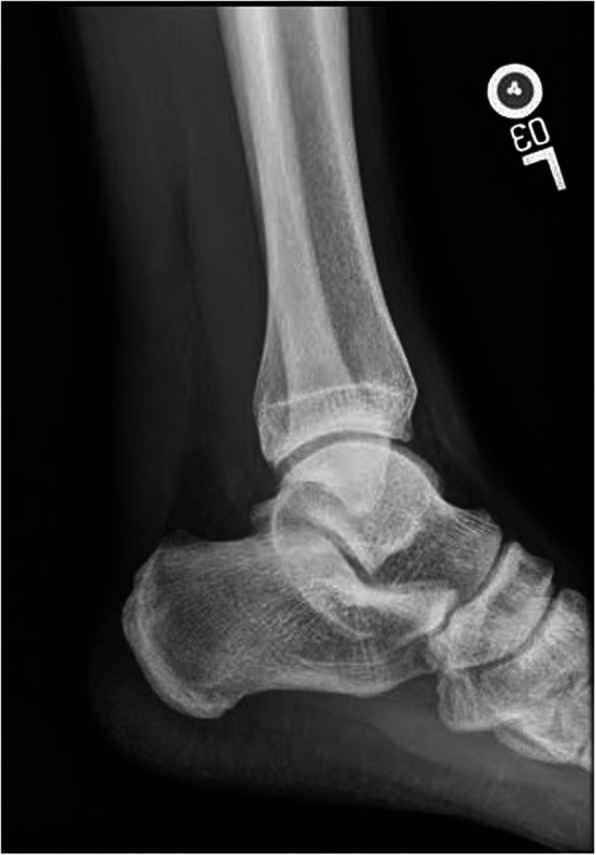
Lateral Radiograph of a left ankle demonstrating disruption of Kager’s triangle

With the use of a web-based survey system, a hypothetical case study was created consisting of a patient with an acute Achilles tendon rupture (Fig. 1). The patient was a 37-year-old male who felt a “pop” in the posterior leg while playing weekend basketball and no history of antecedent Achilles pain. Age chosen as an average from literature of both US Army data and US general population studies on Achilles tendon ruptures [16, 17]. His physical exam was remarkable for a palpable gap 4 cm proximal to the Achilles insertion on the calcaneus and an abnormal Thompson’s test [18]. A lateral non-weight bearing radiograph of the ankle demonstrated disruption of Kager’s fat pad (Fig. 2). Respondents were asked if they would obtain further imaging prior to treatment (Magnetic Resonance Imaging /MRI, Ultrasound, or other), how they would definitively treat the patient (non-operative or operative), method of immobilization, use of deep venous thrombosis (DVT) chemoprophylaxis, and time to release to activities. Subsequent questions depended on responses to prior questions. For example, if a respondent did not require any further imaging, they were never provided with an option for MRI or Ultrasound and the opposite being true if they did require further imaging. The options for chemoprophylaxis were Aspirin, Heparin, Enoxaparin Sodium, Coumadin, Rivaroxaban, Apixaban and “Other”. See Additional file 1: Appendix A for a copy of the survey.

Correlation analysis compared survey data on practice preferences, specifically need for further imaging, method of treatment, and post-operative management, with respondent’s collected demographic data. One-way ANOVA and chi-square analysis performed on respondent data using SPSS software, (SPSS, Chicago, IL, USA), with a *p*-value of < 0.05 for statistical significance.

## Results

Respondent characteristic data are listed in Table 1. Respondent data is summarized in Table 2. Analysis of treatment algorithm for respondents who chose operative management, showed no statistically significant difference in 1) need of further imaging 2) time to surgery 3) technique used 4) post-operative immobilization 5) length of immobilization 6) weight-bearing protocol and 7) time to release to running (*p* > 0.05).

**Table 1:** Basic Respondent Demographics

Data	N (%)
Fellowship Trained	31 (50%)
< 5 years in practice since residency training	34 (55%)
Academic Setting (MEDCEN)	27 (43%)
Community Setting (MEDDAC)	45 (57%)

**Table 2:** Respondent Data

		Percentage answered "Yes"
**General Characteristics/Questions:**	Treat without further imaging	82% (51/62)
	Operative management	62% (38/62)
	Return to running within 4-6 months	71% (44/62)
	Chemoprophyalxis while undergoing treatment	40% (24/62)
**Respondents who treated with Operative management**	Post-op cast/splint in plantarflexion	82% (32/38)
	Immobilize for <3 weeks	54% (20/38)
	Non-weight bearing 0-3 weeks	71% (27/38)
	Traditional open repair	60% (23/38)
	Chemoprophyalxis while immobilized and non-weight bearing	51% (14/27)
**Respondents who treated with Non-operative management**	Accelerated Rehabilitation protocol	95% (22/24)
	Initial immobilization in splint/cast in plantarflexion	71% (17/24)
	0-3 weeks of immobilization	46% (11/24)
	0-3 weeks of non-weight bearing	62% (15/24)
	Chemoprophylaxis while undergoing treatment	25% (6/24)

**Fig. 3:**
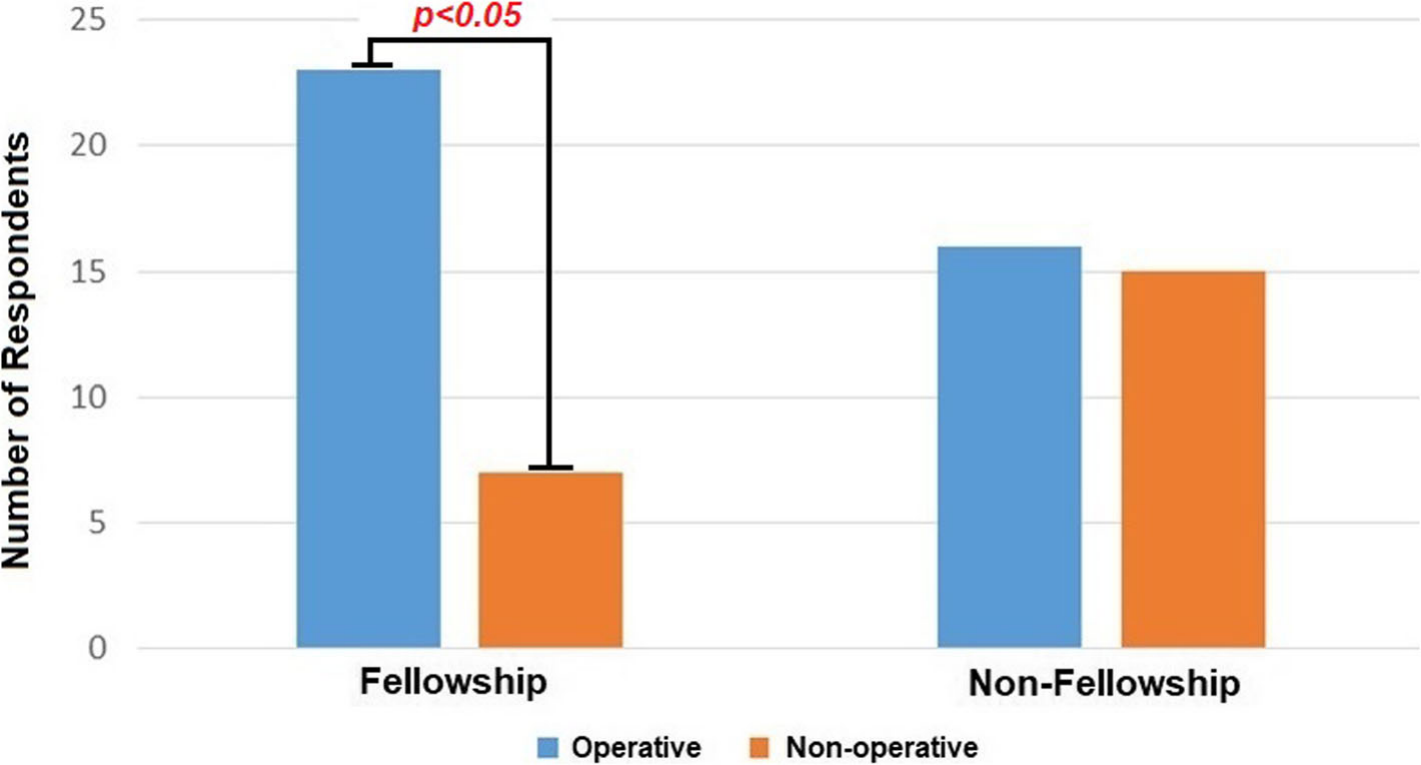
Management stratified by fellowship training. Operative management found to be associated with fellowship training (*p* < 0.05). Legend: Blue = Operative, Orange = Non-Operative

**Fig. 4:**
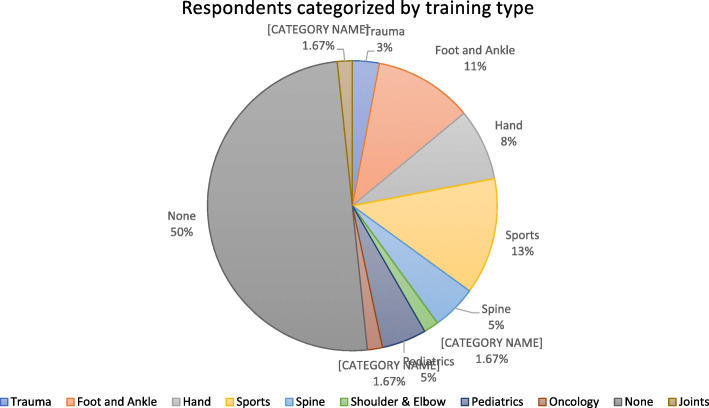
Respondents categorized by training type. Legend: Sky Blue: Foot and ankle; Red = Hand; Dark green = Joints, Orange = none; Yellow = Oncology; Turquoise = Pediatrics; Pink = Shoulder & Elbow; Purple = Spine; Light green = Sports; Dark Blue = Trauma

Fellowship training correlated with operative management (*p* < 0.05, power = 0.905, Fig. 3), with a subgroup analysis showing Hand and Sports surgeons choosing operative management. Fellowship training subgroup analysis is demonstrated in Fig. 4. There was no correlation between years in practice nor practice setting, and operative management (*p* > 0.05), however post-hoc analysis showed a power of only 0.05. Among those who chose operative management, years in practice did correlate with operative technique (*p* < 0.05), with younger surgeons using mini-open/minimally invasive/percutaneous techniques.

## Discussion

This survey study provides a snapshot of the treatment preferences of acute Achilles tendon ruptures among some US Army orthopaedic surgeons. We found that 62% of respondent Army orthopaedic surgeons would treat an acute Achilles rupture with operative management, despite the current trend towards non-operative management for Achilles ruptures [19]. Recently Maffulli et al. found similar outcomes when treating acute and sub-acute (14–30 days) Achilles tendon rupture with a minimally invasive approach [20]. This data would suggest that maybe Army orthopaedic surgeons do not need to be treating so many soldiers in an acute setting (< 1 week), as seen in our respondent population. In Maffulli’s study the sub-acute population was not provided with any initial treatment (splint immobilization, etc) and likely seeked treatment secondary to the pathology causing issue with quality of life. In the military setting, this delay would take a soldier out of their duty prior to surgical management, and then rehab for roughly 4–6 months to get back to full return to duty. In the setting of a missed diagnosis that is caught within 30 days, Maffulli’s study provides adequate evidence for repair that will have success similar to acute repairs, however in the author’s opinion it does not change the decision to operative on a soldier who is seen acutely with an Achilles tendon rupture.

Renninger et al. compared non-operative and operative management of Achilles ruptures in an active duty population, and showed no difference in functional outcomes, complications, and rate of return to duty. However, they found operative treatment correlated with earlier return to duty by roughly 1.5 months [16]. Distinction should be made between “rate of return to duty” and “earlier return to duty”, as one relates to the percentage of patients who returned to active duty. In contrast, “earlier return to duty” corresponds to earlier time between surgery and return to duty. Though our study did not look at the respondent’s rationale for their management style, in the authors’ experience, delayed assessment and delayed immobilization with Soldiers injured overseas, lack of controlled physical therapy protocols, and the transient nature of both Soldier and surgeons are less salient factors that may contribute to decisions. Despite these limitations in the active duty population, this study group would ideally treat an acute Achilles rupture within 1 week. This is consistent with the recent data presented by Svedman et al., which stated that a shorter time to surgery correlated with better outcomes and lower number of adverse events and best when treated within 48 h of injury had the best outcomes [21].

In 2010, the American Academy of Orthopaedic Surgeons (AAOS) released Clinical Practice Guidelines (CPG) on the diagnosis and treatment of acute Achilles tendon ruptures. The CPG provided 16 recommendation statements about treatment of acute Achilles tendon ruptures, with a stated strength of recommendation. Recommendations were split into “Strong”, “Moderate”, “Limited” or “Consensus” based on the overall strength of the evidence for each statement. Of the 16 statements, only two had “Moderate” and two had “Consensus” recommendations and the remainder were “Inconclusive” or “Limited” evidence. Statements which had multiple Level II or III studies to support the recommendation were given a grade of “moderate”. The two “moderate” recommendation were about the use of limited (< 2 weeks) of protected weight bearing and to allow mobilization by 2–4 weeks, both in relation to operative management of acute Achilles tendon ruptures. Surveyed surgeons followed with the moderate recommendations for post-operative protected device and immobilization post-operative for < 2 weeks.

The grading of “consensus” relates to the opinion of the work group in the absence of reliable evidence. The work group’s opinion was that history and physical exam, to include special tests (Thompson test, Matles test, palpable gap, decreased plantarflexion strength) should be adequate to provide a diagnosis for an acute Achilles tendon rupture. In addition, the group’s opinion is that caution must be taken when treating patients with medical co-morbidities with operative management [18]. In our study, 82 % of respondent would treat without further imaging and inherent to the military population, patients tend to be young and healthy, which corresponds with the “consensus” recommendation by the AAOS CPG.

The fear of delayed diagnosis or missed diagnosis in > 20% of cases [22], may lead providers to second guess their history or physical exam, and rather look to advanced imaging as a modality to confirm the diagnosis of an acute Achilles tendon rupture. However, the AAOS work-group found the need for advanced imaging to be an “inconclusive” recommendation. Radiographs provide detail about bony anatomy and may show a disruption of Kager’s triangle/fat pad [23–26], but provide little details about the structural integrity of the Achilles tendon. However, in instances of an acute Achilles sleeve avulsion, when the tendon ruptures distally from its calcaneal insertion as a continuous “sleeve”, a radiograph is integral in surgical planning and management [27]. MRI will provide significant more detail about the locations and nature of a rupture, however an MRI is time consuming, expensive, and can lead to treatment delays, especially when physical examination is more sensitive than MRI [28].

The CPG was released in 2010 and does not take into consideration the more recent data, which could make an impact on the practice of orthopaedic surgeons in the future. For example, there was a slight preference for the traditional open repair (59%). However, when considering years in practice, younger surgeons preferred minimally invasive techniques. Exposure to recent literature demonstrating good to excellent results with low complication rates may influence this decision making [5, 29].

Rates of DVT with Achilles tendon ruptures range from as low as 0.43% [30] to upwards of 34% [31]. Our study population’s lack of DVT chemoprophylaxis after Achilles ruptures (62%) may reflect the lack of consensus literature. Though the incidence of asymptomatic and symptomatic deep venous thrombosis is high after Achilles tendon rupture, there is a need to define the possible benefit of thromboprophylaxis considering data suggesting no difference in DVT frequency between operative and non-operative management [30–33]. Lapidus et al. studied 91 patients treated surgically for Achilles tendon ruptures, and found no difference in the rate of DVTs despite randomized to chemoprophylaxis or a placebo [34].

Part of the confusion of rates of DVT with Achilles ruptures may in fact be due to immobilization. With the advent of the Willits protocol [6] and accelerated rehab protocols post-op, the necessity of lengthy immobilization has decreased. The CPG concluded < 4 weeks of immobilization to be a “moderate” recommendation. Prior to 2010, practitioners treated Achilles tendon ruptures with 6 weeks of cast/splint immobilization. Prior studies have shown that long cast/splint immobilization in the setting of lower extremity injury is a risk factor of DVT/PE [35, 36]. Healy et al. found a rate of DVT of 6.3% with patients treated with 6 weeks of cast immobilization for Achilles tendon ruptures, which was higher than the 2.5% of patients treated with Low-molecular weight heparin (Lovenox) prophylaxis [37]. The study’s conclusion was that consideration should be taken for long term immobilization with Achilles tendon ruptures and DVT prophylaxis. However, since their study many studies, like Lapidus et al., shows no difference in DVT rates despite prophylaxis. In addition, current guidance is for limited immobilization, which should help decrease the incidence of DVT with Achilles tendon ruptures. It is the author’s opinion to treat both operative and non-operative Achilles tendon ruptures with a 2-week splint immobilization and not treat with prophylactic DVT chemoprophylaxis.

This study has multiple limitations. First, the study method employed a hypothetical case, rather than an actual patient, so the bias or preference of the patient was not offered as a factor to assist the surgeon with his/her treatment decision. Though not directly questioned in this study, some surgeons may view both operative and non-operative management as reasonable options and allow patients to dictate their management after discussion of the risks and benefits. Second, the sample size of surveyed surgeons was small and pre-study power analysis was not performed. It is unclear if the lack of statistical findings in this study is from a Type II error due to low sample size. The response rate was only 43% and limited to Army orthopaedic surgeons, so our results may not be generalizable to other groups of surgeons. This allows for a higher chance for sampling bias within the study. Third, as a survey study, we cannot comment directly on clinical outcomes. Fourth, this data provides only a snapshot of the current trend which could change year to year, particularly as new literature continues to be published on the topic.

## Conclusions

US Army orthopaedic surgeons, when presented with an acute Achilles tendon rupture, tend towards operative management. However, with a rate of 62%, this only shows a snapshot rather than a trend. In addition, the percentage shows that non-operative modalities are still used in active patients, though success rates were not assessed. In the author’s opinion operative management allows for fulfillment of the desire to return a soldier to duty “faster” and have better control on the management of the patient.

## Additional file

**Additional file 1: Appendix A**. Sample of Survey. A sample of the survey questionnaire provided to respondents.

## Data Availability

The datasets used and/or analyzed during the current study are available from the corresponding author on reasonable request.

## Abbreviations

DVT: Deep Vein Thrombosis
PE: Pulmonary Embolism
US: Ultrasound
MRI: Magnetic Resonance Imaging
AAOS: American Academy of Orthopaedic Surgeons
CPG: Clinical Practice Guidelines
SPSS: Statistical Product and Service Solutions
CAM: Controlled Ankle Motion
