# Supplementary material for: Management of acute Achilles tendon ruptures: a survey of Army orthopaedic surgeons
Source: BMC Musculoskelet Disord. 2021 Mar 11;22:267. doi: 10.1186/s12891-021-04121-y (PMC7953566; doi:10.1186/s12891-021-04121-y)
Supplement: Supplementary file 1 — Additional file 1: Appendix A. Sample of Survey. A sample of the survey questionnaire provided to respondents. [file 12891_2021_4121_MOESM1_ESM.docx]

| 1. **Would you obtain further imaging?** - Yes -No | | | | |
| --- | --- | --- | --- | --- |
| **No** | | **Yes** | | |
|  | | 1. What further imaging would you obtain? - Magnetic Resonance Imaging - High-Resolution Ultrasonography - Other (please specify) | | |
|  |  | 2a – Further diagnostic imaging shows a full-thickness mid-substance Achilles tendon rupture | | |
| 1. **How would you treat this patient**? - Operative management - Non-operative management - Other | | | | |
| **Operative management** | **Non-operative management** | | **Other** |  |
| 1. **How soon would you prefer to take this** patient to surgery? - Within 24 hours - Within 3 days - Within 1 week - Within 2 weeks - Other | 4**. What would be your initial method of immobilization?** - None - Splint/Cast in plantarflexion - CAM Boot with heel lift - Ankle Stabilizing Orthosis (ASO) brace - Other | | 4**. What would be your initial method of immobilization?** - None - Splint/Cast in plantarflexion - CAM Boot with heel lift - Ankle Stabilizing Orthosis (ASO) brace - Other |  |
| 1. **Technique for management?** - Traditional open repair - Mini-open/Percutaneous repair (i.e. PARS/Achillon) - Midsubstance repair to calcaneus (i.e. Speed Bridge) - Other | 5. **IF you immobilize, how long do you splint/cast/boot?** - 0-3 weeks - 4-6 weeks - 7-10 weeks - >11 weeks - Other | | 5. **Do you use an accelerated rehab protocol?** -Yes -No |  |
| 1. **What would be your method of immediate post-operative immobilization?** - None - CAM Boot with heel lift - Splint/Cast in plantarflexion - Ankle Stabilizing Orthosis (ASO) brace - Other | 6. **What is your weight-bearing protocol?** - NWB 0-3 weeks - NWB 4-6 weeks - NWB 7-10 weeks - NWB >11 weeks - Other | | 6. **What is your weight-bearing protocol?** - NWB 0-3 weeks - NWB 4-6 weeks - NWB 7-10 weeks - NWB >11 weeks - Other |  |
| 1. **How long do you immobilize in a splint/cast/boot?** - 0-3 weeks - 4-6 weeks - 7-10 weeks - >11 weeks - Other | 7. **Do you use an accelerated rehab protocol?** -Yes -No | | 7. **IF you immobilize, how long do you splint/cast/boot?** - 0-3 weeks - 4-6 weeks - 7-10 weeks - >11 weeks - Other |  |
| 1. **Post-operative weight-bearing protocol:** - Immediate weight-bearing as tolerated - NWB 0-3 weeks - NWB 4-6 weeks - NWB 7-10 weeks - NWB >11 weeks - Other |  | |  |  |
| 9. **When do you release patients to return to running?** - 6 weeks - 3 months - 4 months - 6 months - Other | | | | |
| 10. **Do you routinely use DVT chemoprophylaxis for acute Achilles tendon ruptures?** -Yes -No | | | | |
| **Yes** | | **No** | | |
| 11. **What type of DVT chemoprophylaxis do you use?** - Aspirin - Heparin - Lovenox (Enoxaparin sodium) - Warfarin (Coumadin) - Xeralto (Rivaroxaban) - Eliquis (Apixaban) | |  | | |
| 12. **Years in practice since residency training?** - <5 years - 5-9 years - 10-14 years - 15-25 years - >26 years | | | | |
| 13. **Work Setting?** - Military Non-Academic (no residency program) - Military Academic (residency program) - Non-Military Non-academic (no residency program) - Non-Military Academic (residency program) | | | | |
| 14. **Have you completed fellowship training?** - None  - Yes, Foot & Ankle - Yes, Trauma - Yes, Joints - Yes, Spine - Yes, Shoulder & Elbow - Yes, Hand - Yes, Sports - Yes, Pediatrics - Yes, Oncology - Other | | | | |
